# Clinical characteristics and variables associated with COVID-19 morbidity and mortality in Luanda, Angola, in the first year of the pandemic

**DOI:** 10.4314/ahs.v23i4.9

**Published:** 2023-12

**Authors:** Margarete LT Arrais, Welwitschia A F Dias, Maura P A Silva, Luquenia E S Neto, Naiol M F Pedro, Sónia F I Jungo, Avelina R C Miguel, Elsa M V Fortes-Gabriel, Cruz S Sebastião, Jorge M R Gama, Miguel D Brito

**Affiliations:** 1 Department of Pulmonology, Military Hospital, Luanda, Angola; 2 Centro de Investigação em Saúde de Angola (CISA), Caxito, Bengo, Angola; 3 Instituto Superior Técnico Militar (ISTM), Luanda, Angola; 4 Instituto Nacional de Investigação em Saúde (INIS), Luanda, Angola; 5 Instituto de Ciências da Saúde (ICISA), Universidade Agostinho Neto (UAN), Luanda, Angola; 6 Center of Mathematics and Applications, Faculty of Sciences, University of Beira Interior, Covilhã, Portugal; 7 Health and Technology Research Center (H&TRC), Escola Superior de Tecnologia da Saúde de Lisboa, Instituto Politécnico de Lisboa, Portugal

**Keywords:** Angola, COVID-19, morbidity, mortality, variables associated

## Abstract

**Background:**

The impact of SARS-CoV-2 infection in Africa is still unclear. In comparison to Europe and North America, morbidity and death rates are lower. Several factors have been proposed, including geographical variation in virus impact, environmental factors, differences in age distribution, and the impact of infectious diseases such as malaria, HIV infection and tuberculosis.

**Objectives:**

We investigated the clinical characteristics and putative determinants linked with COVID-19 in Angolan patients.

**Methods:**

Cross-sectional study undertaken at Military Hospital, Luanda, from March 2020 to March 2021. The survey collected sociodemographic and clinical information.

**Results:**

The sample included 1,683 patients aged ≥18 years, 64% men, with mean age of 46.3 years. SARS-CoV-2 was positive in 39% of the cases with RT-PCR. Patients ≥46 years with a level of education of ≥12 years had a considerably higher likelihood of testing positive. About 58% of positive patients had at least one comorbidity, of which hypertension and Diabetes were associated with SARS-CoV-2 infection. HIV and pulmonary TB were putative protective factors. About 14% of positive patients died. Most deaths occurred in patients ≥46 years, with less education and unemployed. Working as a healthcare practitioner was linked to a protective effect. Malignant diseases were the most common comorbidities associated with death.

**Conclusions:**

We identified putative factors related to SARS-CoV-2 infection and mortality. HIV and TB were protective and not associated with mortality. Further study with a broader scope should be conducted to explain the main features related to COVID-19 mortality in Angola.

## Introduction

COVID-19, coronavirus disease 2 related to severe acute respiratory syndrome (SARS-CoV-2), rapidly spread across all continents, exponentially increasing the number of infected individuals and thousands of deaths world-wide.^1^ This initial outbreak gave rise to a pandemic declared by WHO on 11 March 2020.^2^

In Angola, the first two imported cases were reported in March 2020, in Luanda capital of the country, and the epicenter of the pandemic.^3^ From March 2020 to March 2021, a total of 22,311 cases and 537 deaths due to COVID-19 were reported.^3^

To understand the impact of COVID-19 in Angola, some studies reported putative factors related to morbidity, severity, and mortality due to COVID-19, including age over 40 years, symptoms such as cough, fever, gastrointestinal symptoms, and asthenia, as well as comorbidities such as hypertension, diabetes mellitus, and chronic kidney disease.^4^

Since there are few studies conducted in African countries, we proposed to study clinical characteristics and variables associated with COVID-19 in a tertiary-level hospital in Luanda, the capital city of Angola, during the first year of the COVID-19 pandemic.

## Methods

### Study area

This study was carried out at the Military Hospital in Luanda, the capital city of Angola, a sub-Saharan African country, with an estimated population of about 35 million inhabitants.^5^ Luanda is the most populous province, accounting for around 27% of the country's total population.^5^ Military Hospital is a tertiary-level hospital with 270 beds in an urban setting, ready to care for patients with clinical suspicion of COVID-19, diagnostic confirmation using Real-Time Polymerase Chain Reaction (RT PCR) assay, and treatment of moderate, severe, and critical cases.^6^

### Study design

A cross-sectional study was conducted with all patients who were admitted to the emergency services and admitted to the hospital wards with a clinical history suggestive of COVID-19 from March 2020 to March 2021.

### Study population and data collection

Only epidemiological surveys of patients who presented to emergency services with symptoms/signs indicative of SARS-CoV-2 infection were chosen from the 4,279 epidemiological surveys for COVID-19 conducted by the Angolan Ministry of Health / National Directorate of Public Health (MINSA/DNSP).^7^ We removed 285 persons with missing data, and 2,311 persons were excluded because they were asymptomatic and had SARS-CoV-2 RT PCR screening (risk contacts, medical procedures, surgeries among others). A total of 1,683 patients aged ≥18 years old completed the eligibility criteria and were enrolled.

Data were extracted from the clinical processes and as well as supplemented by face-to-face interviews with patients and immediate family members of the deceased persons. The survey includes sociodemographic data (gender, age, area of residence, education level/occupation), date of onset of symptoms, date and reason for hospital admission, symptoms, signs, pre-existing conditions, and comorbidities as well as the clinical outcome of the patients.

Laboratory findings (blood count, creatinine, and glucose) were obtained via patient clinical processes and hospital computer system records. The images of chest computed tomography scan (chest CT scan) were accessed by the hospital computer system and reported by two imaging specialists trained for this purpose, who used the classification proposed by the Radiological Society of North America (RSNA) that classifies lung lesions as typical, atypical and very atypical^8^ and the CO-RADS classification COVID working group of the Dutch Radiological Society, that based on CT findings, the level of suspicion of SARS-CoV-2 infection is classified from very low or CO-RADS 1 to very high or CO-RADS 5 and the severity and stage of the disease are determined according to the comorbidities and the differential diagnosis.^9^

### Case definition

Cases were considered as Suspect, probable, Confirmed, and Death by COVID-19. Suspect of COVID-19 was any person presenting with acute fever and cough or acute onset of any of three or more of the following signs and symptoms such as fever, cough, (weakness and weariness), headache, myalgia, sore throat, runny nose, dyspnea (anorexia, nausea and vomiting), diarrhea, altered mental status and epidemiological data such as residing or working in an area at high risk of transmission of the virus, reside or travel to an area with community transmission within 14 days prior to the onset of signs and symptoms, or work in health facilities and be part of households with confirmed COVID-19, within 14 days prior to the onset of signs and symptoms; Any patient with a severe acute respiratory infection, with fever, cough, starting in the last 10 days and requiring hospitalization.^10^ Patient with probable COVID-19 was any suspect of COVID-19, which presents chest images showing typical lesions of COVID-19; a person with recent onset of anosmia or ageusia, in the absence of any other identified cause.^10^ A confirmed of COVID-19 was every suspected or probable COVID-19 patient, whose confirmatory laboratory diagnosis was obtained by identifying SARS-CoV-2 virus RNA in nasopharynx samples, whose methodology is based on polymerase chain reaction with reverse transcription of viral RNA and real-time amplification reaction (RT PCR).^10^ A death by COVID-19 was all deceased by clinically compatible disease in a confirmed patient of COVID-19.^10^

### Ethical considerations

This study was approved by the Ethics Committee of the Military Hospital Luanda (No 1970/DG/HMP/IS/20) and by the Ethics Committee of the Angolan Ministry of Health (No 04/2021). Participants and relatives of the deceased were informed about the study and gave their verbal agreement to participate. All the data related to the study were anonymized, ensuring the confidentiality of the participants.

### Statistical methods

The data were analysed using the statistical program SPSS, version 28 (IBM Corp, Armonk, NY, USA). Categorical variables were described with frequencies and percentages. The quantitative variables were described with mean, standard deviation (SD), median, and 25th and 75th percentiles. All associations between each of the variables of interest and other variables were established with the use of the chi-square test, Fisher's exact test (when the first was not appropriated), or logistic regression, which estimated their respective odds ratios (OR). The adjusted OR's (aOR) for each variable of interest were estimated using a forward variable selection method, with 5% and 10% significant levels to included and excluded a variable in the model, respectively. P<0.05 was considered statistically significant.

## Results

All 1,683 patients aged 18 years or older, were from urban regions, with a mean age of 46±15 years. Most of them aged 46 to 64 years, had an education level between 9 and 12 years, and were active workers. Of the 1,683 patients, 39.4% had a positive RT PCR test for SARS-CoV-2. Patients aged 46 years or older and with a higher level of education had a significantly higher chance of testing positive for SARS-CoV-2 with RT PCR, and the main symptoms that were significantly related (aOR) with RT PCR positivity were cough, fever, weariness, sore throat, malaise, hypoxemia, anorexia and anosmia or hyposmia (Table 1). The time between the beginning of symptoms and hospitalization was generally 6 days (6.05±8.31) for those who tested positive and 7 days (6.67±12.49) for those who tested negative.

**Table 1:** Baseline and clinical characteristics of the patients (N=1,683)

Patient characteristics	Total N (%)	RT PCR positive N (%)	RT PCR negative N (%)	OR (95% CI)	p value ^a^
Overall	1,683 (100)	663 (39.4)	1,020 (60.6)		
Gender					
Female	603 (35.8)	250 (37.7)	353 (34.6)	1.14 (0.93-1.40)	0.195
Male	1080 (64.2)	413 (62.3)	667 (65.4)	1	
Age, years				1.01 (1.00-1.02)	<0.001
Mean ± SD	46.33±14.60	51.25±14.67	48.28±15.20		
Median (IQR)	46 (35; 57)	50 (36; 60)	45 (35; 55)		
Age range, years					<0.001
18 – 45	678 (40.3)	216 (32.6)	462 (45.3)	1	
46 – 64	734 (43.6)	325 (49.0)	409 (40.1)	1.70 (1.37-2.11)	<0.001
≥ 65	271 (16.1)	122 (18.4)	149 (14.6)	1.75 (1.31-2.34)	<0.001
Education level, years					<0.001
≤ 4	199 (11.8)	59 (8.9)	140 (13.7)	1	
5 – 8	446 (26.5)	131 (19.8)	315 (30.9)	0.99 (0.68-1.42)	0.943
9 – 12	615 (36.5)	231 (34.8)	384 (37.6)	1.43 (1.01-2.02)	0.043
> 12	423 (25.1)	242 (36.5)	181 (17.7)	3.17 (2.21-4.55)	<0.001
Employment					0.697
Unemployed	236 (14.0)	85 (12.8)	151 (14.8)	0.91 (0.53-1.56)	0.717
Employee	1041 (61.9)	415 (62.6)	626 (61.4)	1.07 (0.65-1.74)	0.799
Retired	333 (19.8)	135 (20.4)	198 (19.4)	1.10 (0.65-1.84)	0.730
Student	73 (4.3)	28 (4.2)	45 (4.4)	1	
Healthcare practitioner					
Yes	282 (16.8)	125 (18.9)	157 (15.4)	1.28 (0.99-1.65)	0.064
No	1401 (83.2)	538 (81.1)	863 (84.6)	1	

Symptoms on admission	Total N (%)	RT PCR positive N (%)	RT PCR negative N (%)	aOR (95% CI) ^b^	p value ^a^
Cough	590 (35.1)	302 (45.6)	288 (28.2)	1.59 (1.25-2.02)	<0.001
Fever	501 (29.8)	264 (39.8)	237 (23.2)	2.04 (1.59-2.60)	<0.001
Weariness	489 (29.1)	287 (43.3)	202 (19.8)	2.20 (1.69-2.86)	<0.001
Sore throat	430 (25.5)	205 (30.9)	225 (22.1)	1.94 (1.51-2.51)	<0.001
Malaise	158 (9.4)	109 (16.4)	49 (4.8)	1.83 (1.15-2.92)	0.011
Hypoxemia	110 (6.5)	96 (14.5)	14 (1.4)	5.61 (2.88-10.92)	<0.001
Anorexia	70 (4.2)	54 (8.1)	16 (1.6)	3.06 (1.56 – 5.99)	0.001
Anosmia or hyposmia	48 (2.9)	40 (6.0)	8 (0.8)	13.19 (5.88-29.60)	<0.001

a Wald's test

b Adjusted by all available variables and we use a forward variable selection method with 5% and 10% significant levels to included and excluded a variable in the model, respectively

Likelihood ratio test: p<0.001; Hosmer-Lemeshow test: p=0.966; Cox & Snell R^2^=0.222; Nagelkerke R^2^=0.301; AUC=0.772 (95% CI 0.749 – 0.795, p<0.001); Sensitivity=70.0% and specificity=71.1% (probability cutoff=0.379247); Abbreviations: OR: odds ratio; aOR: adjusted odds ratio; CI: confidence interval

Approximately 58% of SARS-CoV-2 positive patients had at least one comorbidity. SARS-CoV-2 infection was significantly associated with arterial hypertension and other cardiovascular diseases (CVD) and Diabetes Mellitus (DM) while, liver diseases, cerebrovascular diseases, HIV infection, pulmonary tuberculosis and its sequelae were associated with a protective factor (Table 2).

**Table 2:** Comorbidities associated with SARS-CoV-2 infection (N=1,683)

Comorbidities (at least one)	Total N (%)	RT PCR positive N (%)	RT PCR negative N (%)	OR (95% CI)	p value ^a^	aOR(95% CI) ^b^	p value ^a^
Any comorbidity	861 (51.2)	387 (58.4)	474 (46.5)	1.62 (1.33-1.97)	<0.001		
Arterial hypertension and other CVD	630 (37.4)	304 (45.9)	326 (32.0)	1.80 (1.47-2.21)	<0.001	1.75 (1.37-2.23)	<0.001
Diabetes Mellitus	199 (11.8)	112 (16.9)	87 (8.5)	2.18 (1.62-2.94)	<0.001	1.96 (1.38-2.79)	<0.001
Obesity	29 (1.7)	21 (3.2)	8 (0.8)	4.14 (1.82-9.40)	<0.001		
Liver disease	35 (2.1)	6 (0.9)	29 (2.8)	0.31 (0.13-0.76)	0.010	0.20 (0.07-0.57)	0.002
Cerebrovascular disease	86 (5.1)	21 (3.2)	65 (6.4)	0.48 (0.29-0.79)	0.004	0.24 (0.12-0.46)	<0.001
Chronic kidney disease	47 (2.8)	15 (2.3)	32(3.1)	0.72 (0.38-1.33)	0.289		
Malignant diseases	22 (1.3)	9 (1.4)	13 (1.3)	1.07 (0.45-2.51)	0.884		
HIV infection	69 (4.1)	13 (2.0)	56 (5.5)	0.34 (0.19-0.64)	<0.001	0.26 (0.12-0.52)	<0.001
Other immunodeficiencies	16 (1.0)	6 (0.9)	10 (1.0)	0.92 (0.33-2.55)	0.876		
Sickle cell disease	5 (0.3)	1 (0.2)	4 (0.4)	0.38 (0.04-3.44)	0.392		
Malaria and other febrile syndromes	30 (1.8)	14 (2.1)	16 (1.6)	1.35 (0.66-2.79)	0.412		
Pulmonary tuberculosis	34 (2.0)	5 (0.8)	29 (2.8)	0.26 (0.10-0.67)	0.006	0.33 (0.12-0.94)	0.037
Tuberculosis sequelae	30 (1.8)	10 (1.5)	20 (2.0)	0.77 (0.36-1.65)	0.494	0.34 (0.13-0.87)	0.025
Asthma	21 (1.2)	11 (1.7)	10 (1.0)	1.70 (0.72-4.04)	0.226		
Allergic rhinitis	16 (1.0)	11 (1.7)	5 (0.5)	3.43 (1.19-9.90)	0.023		
COPD	10 (0.6)	7 (1.1)	3 (0.3)	3.62 (0.93-14.04)	0.063		
Smoking	6 (0.4)	5 (0.8)	1 (0.1)	7.74 (0.90-66.43)	0.062		

a Wald's test

b Adjusted by all available variables and we use a forward variable selection method with 5% and 10% significant levels to included and excluded a variable in the model, respectively

Likelihood ratio test: p<0.001; Hosmer-Lemeshow test: p=0.966; Cox & Snell R^2^=0.222; Nagelkerke R^2^=0.301; AUC=0.772 (95% CI 0.749 – 0.795, p<0.001); Sensitivity=70.0% and specificity=71.1% (probability cutoff=0.379247); Abbreviations: OR: odds ratio; aOR: adjusted odds ratio; CI: confidence interval. CVD: cardiovascular disease; COPD: chronic obstructive pulmonary disease; HIV: human immunodeficiency virus

Ninety-four (14.2%) of the 663 patients with COVID-19 died. There were no significant differences in mortality across genders. The risk of death increased with age, but the majority of unfavourable clinical outcomes occurred in patients aged 46 years or older, especially aged 65 years or older, in about three and six-fold, respectively, with less schooling in about three-fold, unemployed in about ninefold, while being a healthcare practitioner significantly reduced the risk of mortality. Having any comorbidity was a risk factor for COVID-19-related death and DM, cerebrovascular diseases, and malaria as well other febrile syndromes were the primary comorbidities significantly associated to death, however adjusted OR confirmed only malignant diseases (Table 3). From the time of admission until the time of death, the average hospital stay was four days, with a median of two days.

**Table 3:** Variables associated with COVID-19 mortality (N=663)

Patient characteristics	Total N (%)	Deaths N (%)	Recovered N (%)	OR (95% CI)	p value ^a^	aOR (95% CI) ^b^	p value ^a^
Overall	663 (100)	94 (14.2)	569 (85.8)				
Gender							
Female	250 (37.7)	33 (35.1)	217 (38.1)	1			
Male	413 (62.3)	61 (64.9)	352 (61.9)	1.14 (0.72-1.80)	0.575		
Age, years				1.05 (1.03-1.06)	<0.001		
Mean ± SD	51.25±14.67	58.96±13.18	49.97±14.52				
Median (IQR)	52 (40; 61)	59.5 (53; 66)	51 (39; 60)				
Age range, years					<0.001		
18 – 45	216 (32.6)	12 (12.8)	204 (35.9)	1			
46 – 64	325 (49.0)	51 (54.3)	274 (48.2)	3.16 (1.65-6.09)	<0.001		
≥ 65	122 (18.4)	31 (33.0)	91 (16.0)	5.79 (2.85-11.79)	<0.001		
Education level, years					<0.001		
≤ 4	59 (8.9)	17 (18.1)	42 (7.4)	3.36 (1.68-6.74)	<0.001		
5 – 8	131 (19.8)	25 (26.6)	106 (18.6)	1.96 (1.08-3.56)	0.027		
9 – 12	231 (34.8)	26 (27.7)	205 (36.0)	1.05 (0.59-1.88)	0.859		
> 12	242 (36.5)	26 (27.7)	216 (38.0)	1			
Employment					<0.001		
Unemployed	85 (12.8)	22 (23.4)	63 (11.1)	9.43 (1.21-73.54)	0.032		
Employee	415 (62.6)	42 (44.7)	373 (65.6)	3.04 (0.40-22.95)	0.281		
Retired	135 (20.4)	29 (30.9)	106 (18.6)	7.39 (0.96-56.68)	0.054		
Student	28 (4.2)	1 (1.1)	27 (4.7)	1			
Healthcare practitioner							
Yes	125 (18.9)	3 (3.2)	122 (21.4)	0.12 (0.04-0.39)	<0.001	0.21 (0.05-0.89)	0.034
No	538 (81.1)	91 (96.8)	447 (78.6)	1		1	
Time from Symptom onset date to hospital admission	6.05±8.31	6.38±7.02	5.99±8.51	1.01 (0.98-1.03)	0.676		
Mean ± SD	4 (2; 7)	4 (2; 7)	4 (2; 7)				
Median (IQR)							
Comorbidities (at least one)							
Any comorbidity	387 (58.4)	66 (70.2)	321 (56.4)	1.82 (1.14-2.92)	0.013		
Arterial hypertension and other CVD	304 (45.9)	51 (54.3)	253 (44.5)	1.48 (0.96-2.30)	0.079		
Diabetes Mellitus	112 (16.9)	25 (26.6)	87 (15.3)	2.01 (1.20-3.35)	0.008		
Obesity	21 (3.2)	6 (6.4)	15 (2.6)	2.52 (0.95-6.66)	0.063		
Liver disease	6 (0.9)	2 (2.1)	4 (0.7)	3.07 (0.55-17.01)	0.199		
Cerebrovascular disease	21 (3.2)	8 (8.5)	13 (2.3)	3.98 (1.60-9.88)	0.003		
Chronic kidney disease	15 (2.3)	1 (1.1)	14 (2.5)	0.43 (0.06-3.28)	0.413		
Malignant diseases	9 (1.4)	3 (3.2)	6 (1.1)	3.09 (0.76-12.59)	0.115	12.00 (1.42-101.52)	0.023
HIV infection	13 (2.0)	4 (4.3)	9 (1.6)	2.77 (0.83-9.17	0.096		
Other immunodeficiencies	6 (0.9)	0 (0.0)	6 (1.1)	-	1.000 ^c^		
Sickle cell disease	1 (0.2)	0 (0.0)	1 (0.2)	-	1.000 ^c^		
Malaria and other febrile syndromes	14 (2.1)	5 (5.3)	9 (1.6)	3.50 (1.15-10.67)	0.028		
Pulmonary tuberculosis	5 (0.8)	0 (0.0)	5 (0.9)	-	1.000 ^c^		
Tuberculosis sequelae	10 (1.5)	1 (1.1)	9 (1.6)	0.67 (0.08-5.34)	0.705		
Asthma	11 (1.7)	2 (2.1)	9 (1.6)	1.35 (0.29-6.36)	0.702		
Allergic rhinitis	11 (1.7)	1 (1.1)	10 (1.8)	0.60 (0.08-4.75)	0.629		
COPD	7 (1.1)	1 (1.1)	6 (1.1)	1.01 (0.12-8.48)	0.993		
Smoking	5 (0.8)	2 (2.1)	3 (0.5)	4.10 (0.68-24.88)	0.125		

a Wald's test

b Adjusted by all available variables and we use a forward variable selection method with 5% and 10% significant levels to included and excluded a variable in the model, respectively

Likelihood ratio test: p<0.001; Hosmer-Lemeshow test: p=0.481; Cox & Snell R^2^=0.408; Nagelkerke R^2^=0.731; AUC=0.951 (95% CI 0.925 – 0.977, p<0.001); Sensitivity=92.6% and specificity=94.0% (probability cut-off=0.148324)

c Chi-square or Fisher's exact tests

Abbreviations: OR: odds ratio; aOR: adjusted odds ratio; CI: confidence interval; CVD: cardiovascular disease; COPD: chronic obstructive pulmonary disease; HIV: human immunodeficiency virus

Due to technical and material constraints, only 43.1% of the 663 COVID-19 patients undergo full blood count, glycemia, and creatinine testing. Other hematological, biochemical, and inflammatory laboratory parameters were done in a small number of patients for the same reasons, limiting us from conducting a more in-depth examination. A similar situation happened with a chest CT scan, which was only completed on 14.9% of the patients since the hospital only had one CT machine, which had to be utilized with all patients in the hospital, including those with COVID-19.

Based on these findings, we noticed that leukocytosis, lymphopenia, neutrophilia, anemia, and increased blood glucose and creatinine levels were all associated with mortality; however, adjusted OR confirmed that only lymphopenia, neutrophilia, and hyperglycemia were significantly associated with COVID-19 mortality at about a five-, six-, and two-fold, respectively (Table 4). On the other hand, chest CT scan lesions were mostly typical of COVID-19, corresponding to a 5-point CO RADS classification, in 86.9% and 70.7% patients, respectively, and ground glass opacities with consolidation and crazy paving pattern were significantly associated with the risk of mortality in about five and four-fold, respectively (Table 5).

**Table 4:** Laboratory findings (N=286)

Laboratory findings	Total N (%)	Deaths N (%)	Recovered N (%)	OR (95% CI)	p value ^a^	aOR (95% CI) ^b^	p value ^a^
Overall	286 (100)	70 (24.5)	216 (75.5)				
White blood cell (count, ×10^9^/L) Normal range: 4.0-10.0				1.07 (1.02-1.12)	0.004		
Mean±SD	8.70±8.28	11.64±6.69	7.75±8.53				
Median (IQR) count,	6.9 (4.7; 10.2)	10.4 (7.9; 13.9)	5.9 (4.6; 8.8)				
<4.0 ×10^9^/L	37 (12.9)	3 (4.3)	34 (15.7)	0.43 (0.13-1.50)	0.186		
4.0-10.0 ×10^9^/L	177 (61.9)	30 (42.9)	147 (68.1)	1			
>10.0 ×10^9^/L	72 (25.2)	37 (52.9)	35 (16.2)	5.18 (2.83; 9.50)	<0.001		
Lymphocyte (count, ×10^9^/L) Normal range: 0.8-4.0				1.07 (0.89-1.28)	0.472		
Mean±SD	1.81±5.62	2.87±11.27	1.46±0.73				
Median (IQR)	1.28 (0.91; 1.80)	1.18 (0.74; 1.85)	1.29 (0.96; 1.76)				
<0.8 ×10^9^/L	45 (15.7)	17 (24.3)	28 (13.0)	2.25 (1.14-4.43)	0.019	4.87 (2.13-11.15)	<0.001
0.8-4.0 ×10^9^/L	235 (82.2)	50 (71.4)	185 (85.6)	1		1	
>4.0 ×10^9^/L	6 (2.1)	3 (4.3)	3 (1.4)	3.70 (0.73; 18.89)	0.116	1.92 (0.31-11.79)	0.480
Neutrophil (count, ×10^9^/L) Normal range: 2.0-7.0				1.20 (1.12-1.29)	<0.001		
Mean±SD	6.14±4.56	9.19±5.40	5.15±3.77				
Median (IQR)	5.04 (3.08; 7.62)	8.43 (5.64; 11.05)	4.19 (2.71; 6.51)				
<2.0 ×10^9^/L	27 (9.4)	0 (0.0)	27 (12.5)	-	-	-	-
2.0-7.0 ×10^9^/L	169 (59.1)	27 (38.6)	142 (65.7)	1		1	
>7.0 ×10^9^/L	90 (31.5)	43 (61.4)	47 (21.8)	4.81 (2.69-8.62)	<0.001	5.76 (2.96-11.22)	<0.001
Haemoglobin (g/dl) Normal range: 11.0-16.0				0.91 (0.82-1.02)	0.104		
Mean±SD	11.98±2.35	11.51±2.39	12.13±2.32				
Median (IQR)	12.20 (10.80; 13.60)	11.55 (10.10; 13.10)	12.40 (11.00; 13.70)				
<11.0 g/dl	78 (27.3)	26 (37.1)	52 (24.1)	1.91 (1.07-3.40)	0.030		
11.0-16.0 g/dl	202 (70.6)	42 (60.0)	160 (74.1)	1			
>16.0 g/dl	6 (2.1)	2 (2.9)	4 (1.9)	1.91 (0.34-10.76)	0.466		
Platelet (count, ×10^9^/L) Normal range: 100-300				1.00 (1.00-1.00)	0.933		
Mean±SD	209.21±111.18	209.69±94.61	209.06±116.24				
Median (IQR)	184 (150; 237)	195 (159; 249)	182.5 (147; 232.5)				
<100 ×10^9^/L	24 (8.4)	8 (11.4)	16 (7.4)	1.58 (0.64-3.90)	0.318		
100-300×10^9^/L	225 (78.7)	54 (77.1)	171 (79.2)	1			
>300×10^9^/L	37 (12.9)	8 (11.4)	29 (13.4)	0.87 (0.38-2.02)	0.753		
Glucose (mg/dl) Normal range: 60.0-110.0				1.01 (1.00-1.01)	0.001		
Mean±SD	133.71±81.44	161.27±94.15	114.78±74.96				
Median (IQR)	103.30 (82.5; 155.0)	125.14 (92.0; 210.0)	97.3 (81.1; 144.6)				
<60.0 mg/dl	9 (3.1)	1 (1.4)	8 (3.7)	0.59 (0.07-4.94)	0.630	0.25 (0.03-2.44)	0.233
60.0-110.0 mg/dl	161 (56.3)	28 (40.0)	133 (61.6)	1		1	
>110.0 mg/dl	116 (40.6)	41 (58.6)	75 (34.7)	2.60 (1.49-4.54)	<0.001	2.24 (1.18-4.26)	0.014
Creatinine (mg/dl) Normal range: 0.70-1.40				1.10 (0.93-1.31)	0.255		
Mean±SD	1.50±1.44	1.68±1.54	1.45±1.40				
Median (IQR)	1.14 (0.92; 1.42)	1.31 (1.00; 1.70)	1.10 (0.90; 1.33)				
<0.70 mg/dl	11 (3.8)	2 (2.9)	9 (4.2)	0.85 (1.18-4.09)	0.841		
0.70-1.40 mg/dl	203 (71.0)	42 (60.0)	161 (74.5)	1			
>1.40 mg/dl	72 (25.2)	26 (37.1)	46 (21.3)	2.17 (1.20-3.90)	0.010		

a Wald's test

b Adjusted by all available categorical variables, sex and age and we use a forward variable selection method with 5% and 10% significant levels to included and excluded a variable in the model, respectively

Likelihood ratio test: p<0.001; Hosmer-Lemeshow test: p=0.651; Cox & Snell R^2^=0.206; Nagelkerke R^2^=0.307; AUC=0.797 (95% CI 0.742 – 0.853, p<0.001); Sensitivity=78.6% and specificity=69.0% (probability cut-off=0.223763); Abbreviations: OR: odds ratio; aOR: adjusted odds ratio; CI: confidence interval

**Table 5:** Chest tomography findings (N=99)

Chest CT scan findings	Total N (%)	Deaths N (%)	Recovered N (%)	OR (95% CI)	p value ^a^	aOR (95% CI) ^b^	p value ^a^
Multifocal, peripheral and basal distribution ground glass opacities	65 (86.7)	14 (100.0)	51 (83.6)	-	0.192 ^c^		
Central or peribronchovascular, more apical distribution ground glass opacities	6 (8.0)	0 (0.0)	6 (9.8)	-	0.586 ^c^		
Groundglass opacities and consolidation	25 (33.3)	9 (64.3)	16 (26.2)	5.06 (1.48 – 17.37)	0.010	5.36 (1.47 – 19.48)	0.011
Vascular thickening	31 (41.3)	8 (57.1)	23 (37.7)	2.20 (0.68 – 7.16)	0.189		
Round	5 (6.7)	1 (7.1)	4 (6.6)	1.10 (0.11 – 10.64)	0.937		
Crazy paving pattern	16 (21.3)	6 (42.9)	10 (16.4)	3.83 (1.09 – 13.44)	0.036	4.14 (1.06 – 16.15)	0.041
Reversed halo sign	2 (2.7)	1 (7.1)	1 (1.6)	4.62 (0.27 – 78.67)	0.291		
Lymphadenopathy	1 (1.3)	0 (0.0)	1 (1.6)	-	1.000 ^c^		
Cavitation and calcification	1 (1.3)	1 (7.1)	0 (0.0)	-	0.187 ^c^		
Tree-in-bud	2 (2.7)	1 (7.1)	1 (1.6)	4.62 (0.27 – 78.67)	0.291		
Nodular pattern	5 (6.7)	1 (7.1)	4 (6.6)	1.10 (0.11 – 10.64)	0.937		
Pleural thickening	17 (22.7)	4 (28.6)	13 (21.3)	1.48 (0.40 – 5.48)	0.560		

a Wald's test

b Adjusted by all admissible available variables, sex and age and we use a forward variable selection method with 5% and 10% significant levels to included and excluded a variable in the model, respectively

Likelihood ratio test: p=0.004; Hosmer-Lemeshow test: p=0.915; Cox & Snell R^2^=0.138; Nagelkerke R^2^=0.224; AUC=0.746 (95% CI 0.595 – 0.898, p=0.004); Sensitivity=64.3% and specificity=73.8% (probability cut-off=0.254778)

c Fisher's exact test

Abbreviations: OR: odds ratio; aOR: adjusted odds ratio; CI: confidence interval

## Discussion

This study was carried out at a tertiary-level hospital in Luanda using data from the first year of a pandemic, in which we analysed the clinical characteristics of patients aged 18 years or older who presented to the emergency department with symptoms suggestive of SARS-CoV-2 infection. Patients who tested positive for SARS-CoV-2 were mostly men with a mean age of 51 years. RT PCR positivity was significantly associated with age 46 years or older, schooling level equal or greater than 12 years, and symptoms such as cough, fever, weariness, sore throat, malaise, hypoxemia, anorexia and anosmia or hyposmia. More than half of patients with positive RT PCR had at least one comorbidity, arterial hypertension, other CVD, and DM were related with an increased probability of SARS-CoV-2 infection. HIV infection, pulmonary tuberculosis, and its sequelae, on the other hand, were linked to a protective effect. Death from COVID-19, was substantially linked with age equal or older than 46 years, lower level of education, and being unemployed, but being a healthcare practitioner considerably reduced the risk of COVID-19 mortality. Only malignant diseases were substantially linked with COVID-19 mortality among the major comorbidities. Lymphopenia, neutrophilia, hyperglycemia, ground glass opacities with consolidation, and crazy paving pattern were substantially related to a greater risk of death by COVID-19 based on supplementary testing.

The impact of SARS-CoV-2 infection in Africa is still unknown. In comparison to Europe and North America, morbidity and death rates are lower.^11^ Several factors have been proposed, including geographical variation in virus impact, environmental factors, differences in age distribution, genetic and immunological mechanisms of the host,^12^ limited testing capacity in resource-limited regions, underreporting due to stigma,^13^ and the impact of infectious diseases such as malaria, HIV infection, and tuberculosis.^14^

The mean patient age in our research is lower than in some other studies^15^ but comparable to others.^16^,^17^ However, as other studies have demonstrated,^18^ the risk of mortality increases with age, and our patients did as well. Furthermore, most research has stated that men are more vulnerable to infection and have a higher risk of mortality,^18^ as demonstrated by our findings, and the principal symptoms of our patients are comparable to those described in practically all studies.^17^,^18^

Interestingly, our finding showed that higher education level was significantly associated with a higher risk of being infected with SARS-CoV-2, while the lowest level of education and being unemployed was significantly associated with mortality. This relationship may be associated with socio-economic conditions and different lifestyles, as well as limited access to medical care.^12^,^13^ Interestingly, although we had a significant number of infected healthcare practitioners (19%), being a healthcare practitioner was not a risk factor for SARS-CoV-2 infection, but it did seem to be a protective factor for COVID-19 death. Several studies have found that being a healthcare worker is a risk factor for COVID-19 infection and death^19^,^20^ whereas others have found the reverse.^17^,^21^

These disparities in outcomes might be explained by these healthcare workers' greater awareness, risk perception, and training concerning COVID-19, as well as the availability of biosafety material and general characteristics such as age and pre-existing conditions.^22^ Although we have not studied the specific characteristics of our healthcare practitioners, such as age, comorbidities, COVID-19 training, and use of personal protective equipment, our hospital's “front line” healthcare practitioners are mostly young and without comorbidities, so this is one of the hypotheses to consider.

Approximately 58% of our patients had at least one comorbidity, the most common being arterial hypertension and other CVD, as well as DM, all of which were risk factors for infection and malignant diseases was risk factors for mortality. Evidence suggests that patients' sociodemographic features and behavioral habits, as well as pre-existing health conditions/comorbidities, increase susceptibility to SARS-CoV-2 infection, severity, and risk of mortality,^23^ and our results are in agreement.

In our nation, however, infectious diseases with high incidence rates, such as HIV infection, pulmonary tuberculosis, and its sequelae, have been linked to a protective effect against SARS-CoV-2 infection. Several studies conducted in countries with a high prevalence of infectious diseases showed that HIV infection was related to a higher risk of infection and mortality from COVID-19^24^ which contrasts with our results. However, results similar to ours have been reported by other studies.^15^,^25^ The impact of pulmonary tuberculosis on infection and outcome (severity and mortality) of COVID-19 is unclear. Pulmonary tuberculosis is a health problem in Angola with high rates^26^ and its sequels also. These conditions lowered the likelihood of SARS-CoV-2 infection, but there were no significant changes in COVID-19 mortality. While some studies have reported similar results others showed contradictory results^27^ justified by several factors such as local alterations in lung immunity resulting from pulmonary tuberculosis can also adversely influence host response to SARS-CoV-2 virus. This was demonstrated in an in-vitro study with COVID-19 patients with active pulmonary tuberculosis that had an attenuated interferon-gamma response after stimulation of whole blood with peptides derived from SARS-CoV-2 spike protein, in contrast to a normal response to *Mycobacterium tuberculosis-specific* antigens.^28^ On the other hand, there may be an additive effect of BCG vaccination and prior tuberculosis exposure on overall less COVID-19 severity, and due to the high prevalence of other infectious diseases, such as malaria and HIV infection, medications used to treat them may enhance non-specific immunity or prevent SARS-CoV-2 proliferation or activity.^29^ Regarding HIV infection, pulmonary tuberculosis, and its sequelae, our findings should be interpreted with caution because we did not investigate some specific characteristics of these patients, such as age, degree of immunosuppression, extent and characteristics of lung lesions, BCG vaccination, treatments, and other factors that could influence the findings.

In terms of chronic respiratory disease, we found no statistically significant differences between asthma, allergic rhinitis, COPD, smoking and SARS-CoV-2 infection or mortality. The findings should also be taken with caution since we did not study numerous key characteristics connected to these comorbidities, such as diseases severity and control, treatments performed, tobacco load, and so on. Several research that examined the link between these illnesses and COVID-19 found no link in terms of asthma, but conflicting results in terms of COPD and smoking.^15^,^16^,^18^

Studies carried out during the pandemic have demonstrated the relationship and impact of laboratory biomarkers on the progression of the disease. The haematological and coagulation parameters and increased inflammatory reactions caused by various cytokines and liver enzymes are globally observed phenomenon in COVID-19 patients.^30^

Due to limitations, it was not possible to analyse all these parameters, however, from the analysed parameters our findings are consistent with those of other studies that discovered changes in lymphocytes and neutrophils,^31^ as well as hyperglycemia, which were related to a greater risk of death. Hyperglycemia was found to be a risk factor for severe COVID-19 in previous studies with hospitalized patients, and blood glucose changes may be more likely to occur as a result of severe illness in COVID-19.^32^,^33^ The state of hyperglycemia associated with increased severity and mortality from COVID-19 has been explained that the receptor angiotensin-converting enzyme 2 (ACE2), of SARS-CoV-2 is expressed in islet cells, SARS-CoV-2 can use ACE2 effectively to destroy islet cells, resulting in islet-function damage, hyperglycemia or deterioration of blood glucose in patients with diabetes.^32^ As a result, we believe that hospitalized COVID-19 patients must maintain blood glucose management within a stable and normal range.

Concerning chest CT scan, our results are also similar to previous studies that extensive ground glass opacities with consolidation, so crazy paving pattern are risk factors for COVID-19 mortality,^34^,^35^ as they lead to a rapid deterioration of the patient's clinical condition involving with severe respiratory failure and death. As shown, chest CT scan plays an important role in the approach of COVID-19 patients, not only in the early identification of SARS-CoV-2 pneumonia, but also to stratify patients and evaluate their prognosis. Studies conducted in Africa also concluded that chest CT severity scores can help assess the severity and clinical outcome of COVID-19 and are strongly correlated with laboratory tests.^36^,^37^

There are several limitations to this study. The fact that it is a study based on epidemiological surveys, clinical processes, and computerized patient data may have resulted in several biases, however we attempted to mitigate these elements through interviews with patients. It was carried out at a single facility in an urban region, preventing broader coverage that may have yielded stronger results. Due to resource constraints, only a small number of patients completed laboratory testing and a chest CT scan, and other significant laboratory parameters such as biochemical, hematological, and inflammatory markers could not be analysed. We have not adequately investigated individuals with malaria, HIV infection, pulmonary TB and its sequelae, all of which are common infectious diseases in Angola. Despite these limitations, this study offers vital information on several characteristics linked with COVID-19 infection and mortality in our country with a predominantly youthful population and a high frequency of infectious diseases.

## Conclusions

This study offers critical information for COVID-19 and mortality in Angola. Infection diseases were associated with a protective effect for SARS-CoV-2 infection and were not associated with mortality. Mortality was linked to older age, lesser education, being unemployed, and having a malignant disease. Diagnostic capability should be enhanced for a more effective approach to COVID-19 patients. Future research with a broader breadth and depth should be conducted to better elucidate these issues.
